# Multicenter, prospective feasibility study of Nano-Pulse Stimulation™ technology for the treatment of both nodular and superficial low-risk basal cell carcinoma

**DOI:** 10.3389/fonc.2022.1044694

**Published:** 2022-12-02

**Authors:** Amy S. Ross, Todd Schlesinger, Christopher B. Harmon, Ronald L. Moy, Thomas E. Rohrer, Darius R. Mehregan, Richard Nuccitelli, Lauren Jauregui Johnston, William A. Knape

**Affiliations:** ^1^ PHDermatology, Clearwater, FL, United States; ^2^ Clinical Research Center of the Carolinas, Charleston, SC, United States; ^3^ Surgical Dermatology Group, Vestavia Hills, AL, United States; ^4^ Ronald L. Moy Fincher Chipps Facial Plastics/Dermatology, Beverly Hills, CA, United States; ^5^ Skincare Physicians, Chestnut Hill, MA, United States; ^6^ Department of Dermatology, Wayne State University, Detroit, MI, United States; ^7^ Clinical Studies Dept., Pulse Biosciences, Inc., Hayward, CA, United States

**Keywords:** Nano-Pulse Stimulation therapy, regulated cell death, BCC, basal cell carcinoma, nodular BCC, superficial BCC, low-risk BCC, Nano-Pulse Stimulation

## Abstract

**Background:**

Nano-Pulse Stimulation™ (NPS™) therapy is a new, non-thermal bioelectric modality that applies ultrashort pulses of electric energy to trigger regulated cell death (RCD) in treated tissues. Instead of initiating necrosis by heating or freezing, NPS therapy permeabilizes intracellular organelles to activate the cell’s own self-destruct pathway of programmed or regulated cell death. Unlike cryotherapeutic procedures that can both damage structural tissues and diffuse into the periphery beyond the margins of the lesion, NPS therapy only affects cells within the treated zone leaving surrounding tissue and acellular components unaffected.

**Methods:**

In this study we treated 37 basal cell carcinoma lesions on 30 subjects (NCT04918381). The treated lesions were photographed on 3-, 7-, 14-, 30- and 60-days after treatment. All subjects then underwent surgical excision for histological examination of the treated tissue.

**Results:**

92% of the BCC lesions (34 of 37) showed complete histological clearance of BCC. Histologic analysis of the 3 cases where residual BCC was noted indicated that full energy coverage was not achieved, which could be remedied with an improved treatment guide to standardize and optimize the CellFX^®^ procedure based on NPS technology.

**Conclusion:**

The CellFX procedure was shown to be safe and effective for the treatment of low-risk nodular and superficial BCC lesions.

## Introduction

Every cell in our bodies contains a fail-safe mechanism called regulated or programmed cell death that allows it to self-destruct when it reaches the end of its useful life, encounters a lethal gene mutation or an injury that it is unable to repair (1–3). This pathway can be activated using ultrashort electric pulses during Nano-Pulse Stimulation™ (NPS™) Therapy. Unlike ablation technologies that destroy cells and extracellular tissue by necrosis using extreme heat or cold, NPS therapy is a non-thermal energy modality that triggers the natural cellular self-destruct pathway by initiating a transient permeabilization of the plasma and organelle membranes of targeted cells. This alters the function of internal cellular organelles, including the mitochondria and endoplasmic reticulum (4), without disrupting the extracellular tissue, primarily collagen-rich dermal foundation. Pulse Biosciences is currently marketing the CellFX^®^ System using NPS™ technology to clear benign skin lesions in clinical dermatological practice. Previous published work includes treatments of seborrheic keratosis (5), sebaceous hyperplasia (6) and warts (7). In animal studies, NPS technology has also shown high efficacy in treating a variety of malignant murine tumor types including rat hepatocellular carcinoma as well as mouse breast, fibrosarcoma, squamous cell carcinoma (SCC), pancreatic, lung and melanoma tumors (8–14). A small, first-in-human clinical trial treating BCCs on patients with basal cell nevus syndrome was conducted previously (15).

Basal cell carcinoma (BCC) accounts for 80% of all skin cancers with more than 3.6 million cases diagnosed in the United States each year (16, 17). Surgical removal is the most common therapy using excision, Mohs surgery or curettage and electrodesiccation (18) often resulting in a scar at the excision site. Nano-Pulse Stimulation™ therapy triggers regulated cell death in the lesion and reduces the risk of scarring, making it desirable for the treatment of BCCs on the face or locations not suitable for surgery. We therefore sought to determine if the CellFX System could eliminate BCC lesions with a single treatment as confirmed by histological examination of the excised treated lesion and the safety margin of surrounding tissue.

## Materials and methods

### Study design

This clinical feasibility study was designed to evaluate the safety and effectiveness of the CellFX System in adults for the clearance of low-risk BCC. The study was conducted as a prospective, multicenter, IDE feasibility study. Five clinical sites enrolled 30 subjects under a Central IRB- approved protocol with informed consent over an approximate 4.5-month duration. The study population consisted of adult males and females between 22 and 85 years of age with 1-2 histologically confirmed, primary low-risk, BCC lesions satisfying inclusion and exclusion criteria. Eligible subjects received a single treatment with the CellFX System and were followed at 3, 7, 14, 30 and 60 days post-CellFX treatment. At the 60-day visit, all subjects underwent complete surgical excision of the lesion with 5 mm margins. Each subject returned for 3 additional follow-up visits post-excision at 14 days (suture removal), 30 days and 60 days for a total of 8 study visits.

The primary effectiveness endpoint of complete histological clearance of the target lesion based on microscopic evaluation of hematoxylin and eosin (H&E) stains was evaluated by an independent board-certified dermatopathologist at 60 days post-CellFX treatment. The primary safety endpoint for the rate of occurrence of any serious adverse events related to the CellFX procedure was evaluated throughout the study. Procedural pain assessments were performed at the time of treatment. Standardized photographic images along with live clinical assessment of the treated area were performed at each in-office visit (NCT04918381).

### Methodology

Demographic information and medical history were collected, along with presence and severity of skin effects including erythema, edema, exudate, eschar, peeling scaling, bleeding, ulceration, scarring, and pigmentary changes by the treating investigator and rated on a 5-point scale: None, Mild, Moderate, Moderate to Severe, and Severe.

**Figure 1:**
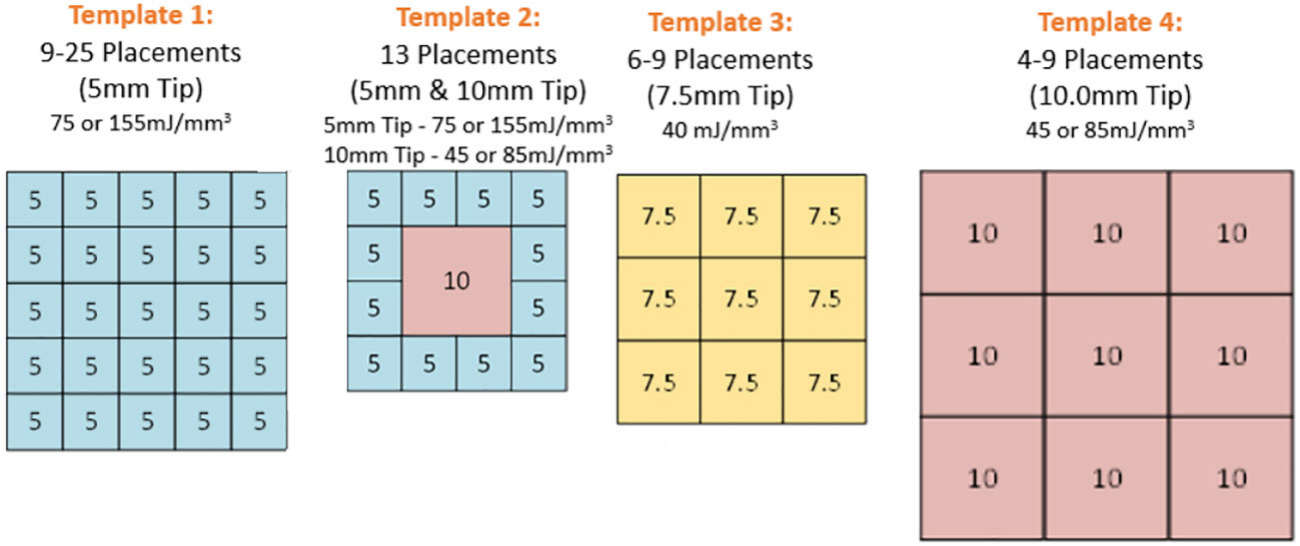
Templates used to guide applicator tip placement over BCCs.

**Figure 2:**
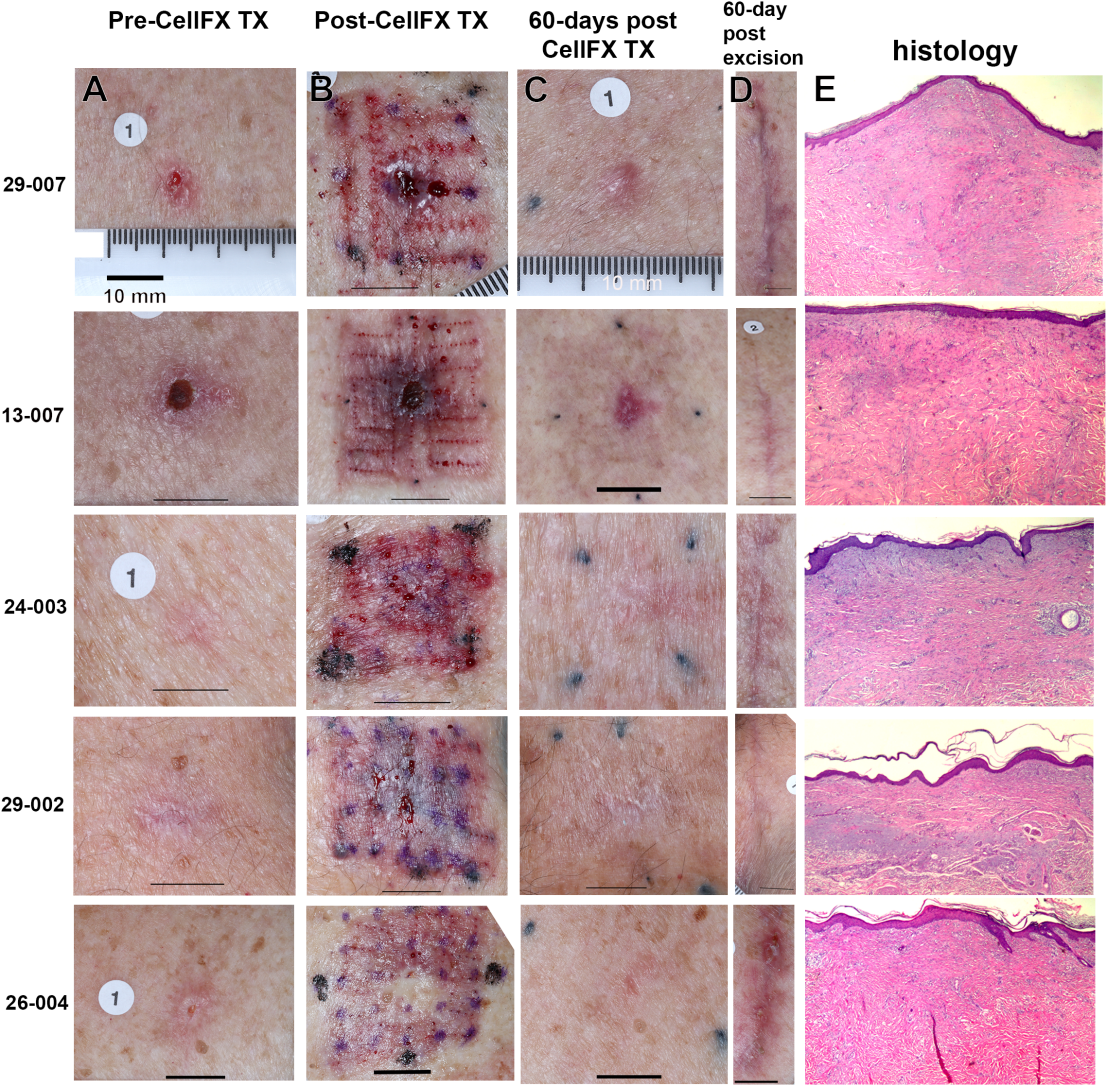
Five BCC lesions treated with the CellFX System and excised after 60 days for histological examination. 29-007 was treated with four placements of the 10 x 10 mm tip; 13-007 was treated with nine placements of the 7.5 x 7.5 mm tip; the remaining three were treated with 9, 12, and 16 placements, respectively, of the 5 x 5 mm tip. **(A)** Photos of lesions before treatment. **(B)** Photos of lesions immediately after treatment. **(C)** Photos of lesions 60 days after treatment. **(D)** The appearance of the skin 60 days post-excision is shown for comparison to the skin appearance 60 days post-CellFX. **(E)** Tissue sections (5-µm thick) stained with H&E all indicate clearance of the BCC. The scale bar in each image represents 10 mm.

One of four clear plastic templates were applied to the skin to guide adjacent placements of the CellFX treatment tip on a grid designed to cover the entire lesion along with a 5mm margin around the lesion (**Figure 1**). Four tattoo dots were applied to mark the treatment zone. Local injected lidocaine (1-2%) was applied to each treatment zone prior to treatment totaling 2-5 ml for the treatment area (**Figure 2**). A single treatment session was performed, consisting of 4-25 treatment cycles applied to the skin *via* microneedle skin surface application with options of 3 different tip sizes. The treatment energies are listed in **Table 1**. Twelve subjects were treated with the 5.0 X 5.0mm tip, 13 subjects were treated with both that tip and a 10.0 X 10.0mm tip, 4 subjects were treated with the 7.5 x 7.5mm tip and 8 subjects were treated with the 10.0 X 10.0mm tip.

**Table 1:** Energies applied for each tip size.

Tip size (length x width)	Energy Density (mJ/mm^3^)
5.0mm X 5.0mm	75 and 155
7.5mm X 7.5mm	40
10.0mm X 10.0mm	45 and 85

### Subjects

Of the 30 subjects enrolled, 53% were female with a mean study age of 65 years (37-81 years). Approximately 97% of the subjects were identified as white and 3% American Indian. 80% of subjects reported prior history of BCC lesions (1-50 lesions) that had been treated with Mohs micrographic surgery (75%), surgical excision (63%), curettage and electrodesiccation (25%), topical 5-fluorouracil (8%) and cryosurgery (8%). Each subject had at least one histologically confirmed, primary low-risk BCC lesion. Most BCC lesions (84%) had a nodular component (68% nodular and 16% superficial and nodular) and the remaining were diagnosed as superficial.

## Results

**Figure 3:**
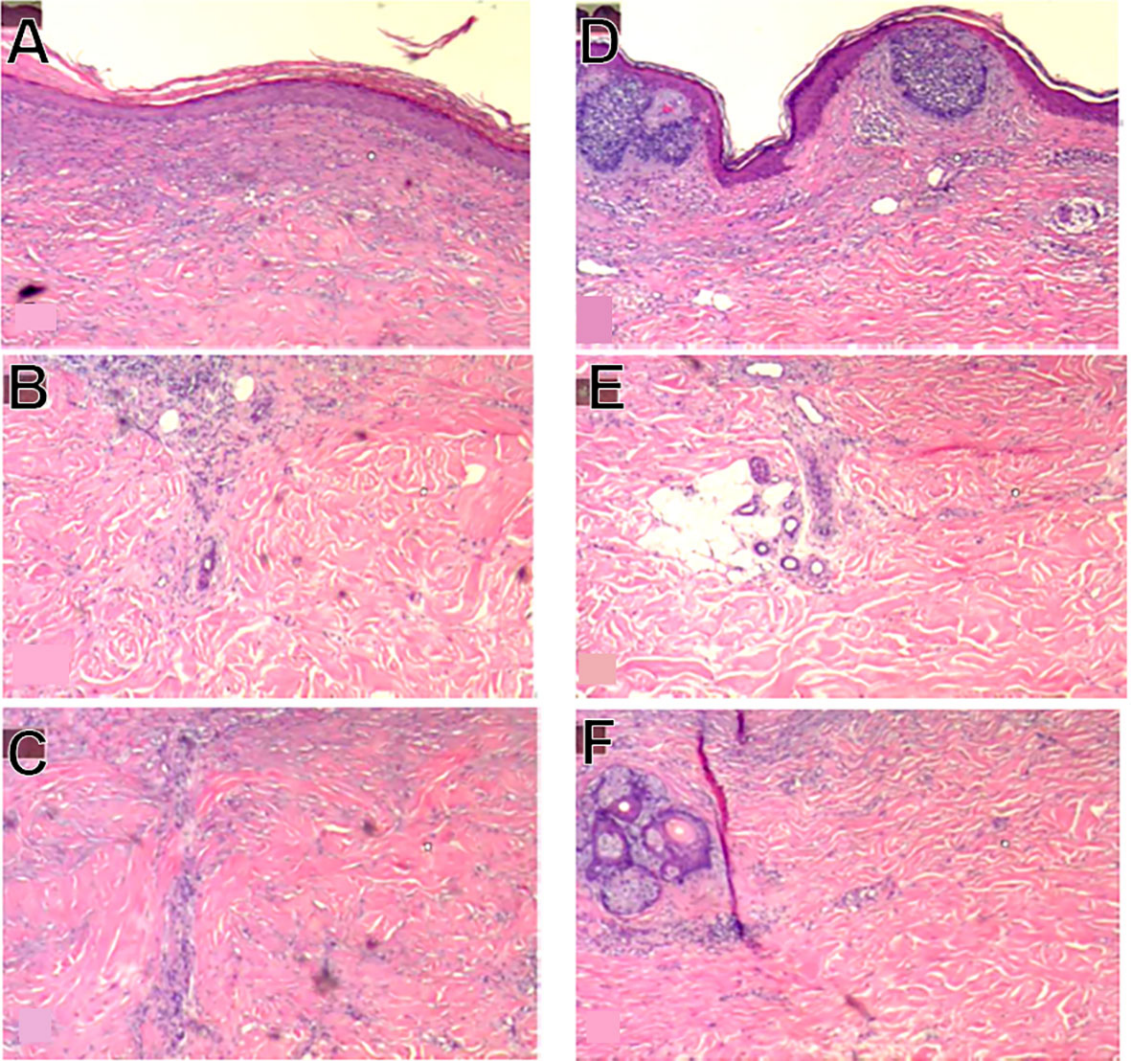
Example of identified treatment zone **(A-C)** and area with residual BCC **(D-F)**. **(A)** shows flattening of the epidermal/dermal junction and mild dermal fibrosis compared to **(D)**. **(B)** shows a sweat duct under the clear treatment zone where the majority of the adnexal structure is destroyed, compared to **(E)** where the sweat duct is located beneath the residual BCC and is completely intact. **(C)** shows presence of thicker collagen in the clear treatment zone where thicker collagen is not observed in **(F)**.

92% of the BCC lesions (34 of 37) showed complete histological clearance of BCC based on examining at least 7 five-micron sections through each lesion stained with H&E (**Figure 2E**). 100% histological clearance was seen with the 5.0 mm tip with less than 20 cycles (10 of 10) and 97% histological clearance was seen for the 5 mm and 10mm tip cohort (32 of 33). The three lesions that showed residual BCC were found to have incomplete energy coverage in one of the adjacent treated areas, based on histological absence of fibrosis just beneath the regenerated dermis, the lack of flattening of the epidermal/dermal junction, or the presence of adnexal structures such as sweat glands normally cleared by CellFX^®^ procedure. **Figure 3** depicts the treated zone from an untreated zone and illustrates the three unsuccessful treatments in this study.

### Safety analysis

There was no incidence of any serious AEs or complications associated with the treatment of BCCs with the CellFX system. There was no evidence of thermal damage in any of the histological specimens and all showed a completely healed epidermis. Underlying nerves and eccrine glands also appeared intact within the excision specimens including underneath the treatment zones.

### NPS procedure and local skin effects

Intralesional injection of lidocaine was effective in managing subject discomfort during the CellFX treatment procedure, with the average subject reported pain rated as mild (2/10 utilizing a standardized 11-point pain scale). The most common skin effects observed 60 days post-CellFX treatment included scarring (84%; 38% at baseline), erythema (68%; 95% at baseline), hyperpigmentation (27%), and scaling (22%) (**Table 2**).

**Table 2:** Local Skin Reactions Post 60-Days (CellFX vs. Standard Surgical Excision).

Local Skin Reaction	Baseline	Rating at 60 Days Post-CellFX	Rating at 60 Days Post-Excision
Scarring	14 (38%)	31 (84%)	35 (95%)
Erythema	35 (95%)	5 (68%)	29 (78%)
Hyperpigmentation	0 (0%)	10 (27%)	7 (19%)
Scaling	7 (19%)	8 (22%)	4 (11%)
Edema	7 (19%)	7 (19%)	3 (8%)
Peeling	6 (16%)	5 (14%)	3 (8%)
Hypopigmentation	3 (8%)	4 (11%)	4 (11%)
Eschar	6 (16%)	3 (8%)	0 (0%)
Ulceration	3 (8%)	3 (8%)	1 (3%)
Exudate	0 (0%)	2 (5%)	0 (0%)
Bleeding	1 (3%)	0 (0%)	0 (0%)

### NPS treatments exhibit minimal scarring

**Figure 4:**
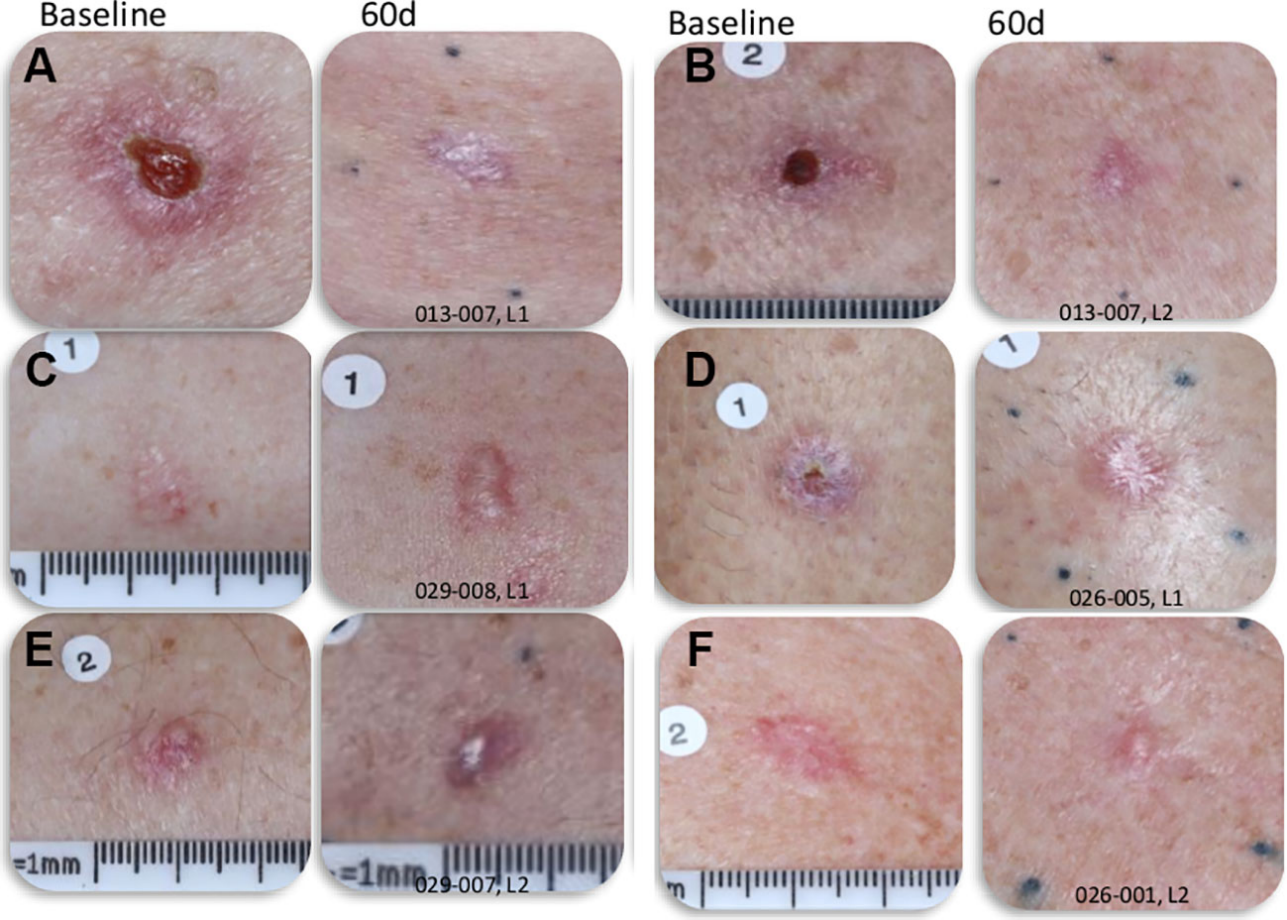
Photographs of BCC lesions before and 60-days post-CellFX Treatment. The scar in the treated lesions correlates well with the site of biopsy that was completed before CellFX treatment and regions outside of the biopsy that were treated with CellFX System showed minimal to no scarring. **(A–F)** each represents a separate lesion shown before and 60d after treatment.

While 84% of the NPS-treated BCC lesions had some scarring at 60 days post-CellFX procedure, a careful comparison of the scar with the pretreatment photo indicated that most scars may have been caused by the pre-treatment biopsy rather than CellFX treatment (**Figure 4**). CellFX treatments covered the lesion and included a 5 mm margin. However, the only scar observed on the 60-day images was correlated with the site of biopsy. Marginal areas surrounding the lesion treated with CellFX System were shown to be free of scar tissue. Investigators rated 95% of lesions as cosmetically acceptable. According to physician evaluation, the CellFX treated area was rated as less likely to scar compared to surgical excision. 89% of CellFX treated areas are expected to look better than curettage and electrodesiccation and 78% are expected to look better than standard surgical excision.

## Discussion

CellFX treatment is extremely effective in clearing BCC lesions. In this clinical feasibility trial, five Mohs surgeons treated 37 lesions with 4 to 25 tip placements, resulting in complete histological clearance when complete energy coverage is applied to the BCC. Moreover, 85% of the histologically clear lesions had a nodular basal cell component which are usually the hardest to clear. Using a single treatment of the CellFX System is extremely efficient and effective for the treatment of low-risk BCC lesions. The cosmesis observed 60-days post treatment indicates minimal scarring compared to surgical removal with no serious adverse events or complications associated with the CellFX Procedure or treatment. Since three lesions resulted in residual BCC, due to minor errors in tip placement, there is a need for providing an improved placement guide to standardize the application of tip placement. There is also potential to explore optimal energy levels to further improve cosmesis. The scarring rated at 60 days post-excision was 95% of the lesions compared to only 84% post-CellFX treatment which were attributed to the original diagnostic biopsy and not the CellFX treatment procedure due to the focal scarring in the area of the BCC lesion without scarring in the surrounding margin of the BCC.

## Conclusion

We conclude that the CellFX System is safe and effective for the treatment of low-risk nodular and superficial BCC and may be an emerging, non-surgical treatment option for the treatment of primary BCCs requiring maximal sparing of tissues, including facial lesions.

## Data availability statement

The original contributions presented in the study are included in the article/supplementary material. Further inquiries can be directed to the corresponding author.

## Ethics statement

The studies involving human participants were reviewed and approved by Advarra Institutional Review Board. The patients/participants provided their written informed consent to participate in this study.

## Author contributions

AR, TS, CH, RM, and TR treated the BCCs in this study. DM was the dermatopathologist who evaluated the histological sections for the presence of BCC. RN, LJ and WK designed the study and wrote the manuscript. All authors contributed to the article and approved the submitted version.

## Funding

Pulse Biosciences funded this study.

## Conflict of interest

Authors RN, LJ and WK are on the payroll of Pulse Biosciences.

The remaining authors declare that the research was conducted in the absence of any commercial or financial relationships that could be construed as a potential conflict of interest.

## Publisher’s note

All claims expressed in this article are solely those of the authors and do not necessarily represent those of their affiliated organizations, or those of the publisher, the editors and the reviewers. Any product that may be evaluated in this article, or claim that may be made by its manufacturer, is not guaranteed or endorsed by the publisher.
